# N-terminally truncated POM121C inhibits HIV-1 replication

**DOI:** 10.1371/journal.pone.0182434

**Published:** 2017-09-05

**Authors:** Hideki Saito, Hiroaki Takeuchi, Takao Masuda, Takeshi Noda, Shoji Yamaoka

**Affiliations:** 1 Department of Molecular Virology, Graduate School of Medicine, Tokyo Medical and Dental University, Bunkyo-ku, Tokyo, Japan; 2 Department of Immunotherapeutics, Tokyo Medical and Dental University, Bunkyo-ku, Tokyo, Japan; 3 Division of Ultrastructural Virology, International Research Center for Infectious Diseases, Institute of Medical Science, The University of Tokyo, Minato-ku, Tokyo, Japan; 4 PRESTO, Japan Science and Technology Agency, Saitama, Japan; 5 Laboratory of Ultrastructural Virology, Institute for Frontier Life and Medical Sciences, Kyoto University, Sakyo-ku, Kyoto, Japan; George Mason University, UNITED STATES

## Abstract

Recent studies have identified host cell factors that regulate early stages of HIV-1 infection including viral cDNA synthesis and orientation of the HIV-1 capsid (CA) core toward the nuclear envelope, but it remains unclear how viral DNA is imported through the nuclear pore and guided to the host chromosomal DNA. Here, we demonstrate that N-terminally truncated POM121C, a component of the nuclear pore complex, blocks HIV-1 infection. This truncated protein is predominantly localized in the cytoplasm, does not bind to CA, does not affect viral cDNA synthesis, reduces the formation of 2-LTR and diminished the amount of integrated proviral DNA. Studies with an HIV-1-murine leukemia virus (MLV) chimeric virus carrying the MLV-derived Gag revealed that Gag is a determinant of this inhibition. Intriguingly, mutational studies have revealed that the blockade by N-terminally-truncated POM121C is closely linked to its binding to importin-β/karyopherin subunit beta 1 (KPNB1). These results indicate that N-terminally-truncated POM121C inhibits HIV-1 infection after completion of reverse transcription and before integration, and suggest an important role for KPNB1 in HIV-1 replication.

## Introduction

Recent genetic approaches have identified a considerable number of host cell factors regulating the replication of human immunodeficiency virus type 1 (HIV-1), including APOBEC3G, TNPO3, NUP358, NUP153, LEDGF/p75, TSG101 and BST-2/Tetherin [1–6]. Like the other lentiviruses, HIV-1 evolved to be able to infect non-dividing cells by delivering its reverse-transcribed DNA into the host cell nucleus through nuclear pore complexes (NPC) [7–13]. A large number of studies has indicated that the HIV-1 capsid (CA) is an essential determinant of HIV-1 infection of non-dividing cells. HIV-1 CA was necessary for infection of non-dividing cells as demonstrated by using chimeric viruses in which the Gag region of HIV-1 had been replaced by that of murine leukemia virus (MLV) [14]. A carboxy-terminally truncated form of mouse cleavage and polyadenylation specificity factor subunit 6 (mCPSF6-358) inhibited HIV-1 infection by preventing nuclear import, and HIV-1 harboring the N74D mutation of CA evaded mCPSF6-358-mediated inhibition, taking an alternative route for nuclear entry [15]. The CA determines which NPC proteins, such as nucleoporin NUP358 and NUP153 or the nuclear transport factor, Transportin 3 (TNPO3), will be employed for crossing the nuclear envelope [16–18].

Nuclear import of host cell proteins is generally mediated by importin family members. Importin-α family proteins mainly recognize nuclear-localizing signals (NLSs) of cargo proteins and importin-βfamily proteins are usually necessary for docking to the NPC and translocation through the nuclear pore [11]. Importin-β/karyopherin subunit beta 1 (KPNB1) protein was shown to bind to and import the HIV-1 proteins Tat and Rev independently of Importin-α [19]. Recent reports showed the TNPO3, a member of the importin-β protein family [20], prevents cleavage and polyadenylation specificity factor subunit 6 (CPSF6)-mediated capsid stabilization in the cytoplasm and contributes to efficient nuclear entry of the CPSF6-associated HIV-1 preintegration complex (PIC) [12, 21]. There have been conflicting reports about the role of an importin-β family member importin-7 in HIV-1 nuclear import [22, 23]. Thus, the roles of multiple host cell factors in HIV-1 nuclear import remain to be further studied.

In the present study, we employed cDNA expression screening to identify an N-terminally truncated form of POM121C which strongly inhibited HIV-1 replication. POM121C is a nucleoporin located in the middle of the nuclear pores which plays an essential role in the formation of NPC [24]. Here, we describe how this truncated protein interferes with HIV-1 replication.

## Materials and methods

### Cells

HEK293 (Invitrogen Corp., Carlsbad, CA), HEK293T (Invitrogen), HeLa (ATCC), and Plat-E packaging cells [25] were propagated in Dulbecco’s modified Eagle’s medium containing 10% fetal bovine serum (FBS) and penicillin-streptomycin. The human T cell lines MT4C5 (kindly provided by Dr. Tetsuro Matano, National Institute of Infectious Diseases, Japan) and Jurkat (kindly provided by Dr. Klaus Strebel, National Institutes of Health, USA) were maintained in complete RPMI 1640 medium supplemented with 10% FBS and penicillin-streptomycin. Phytohemagglutinin (PHA)-activated PBMCs (PHA-PBMCs) (kindly provided by Dr. Rika Ann Furuta, Japanese Red Cross Osaka Blood Center) were cultured in RPMI 1640 containing 10% FBS, penicillin/ streptomycin, and 100 U IL-2 per ml.

### Preparation of virus stocks

HEK293T cells cultured in a 10-cm dish were cotransfected with 8 μg of pNL4-3luc (*env-/nef-*) [26], pNL4-3tk [27], pNL4-3luc CA-N74D [27], pNL4-3luc IN-D116G [28], pLai3ΔenvLuc2 [14], or pMHIV-mMA12CA-Luc [14] plus 2 μg of pHCMV-G (VSV-G) using FuGENE 6 (Promega Corp, Madison, WI) according to the manufacturer’s instructions. Virus supernatant was harvested 48 h post-transfection and filtered through a 0.45 μm-pore syringe filter. Replication-competent HIV-1_NL4-3_ or NL4-3luc-RC virus stocks were prepared by transfection of HeLa cells as previously reported [29]. Viruses were harvested 48 h post-transfection and filtered through a 0.45 μm-pore syringe filter. Titers of the virus stocks were quantified by HIV-1 CA (p24) enzyme-linked immunosorbent assay (ELISA) (ZeptMetrix Corporation, Buffalo, NY) and by determining their reverse transcriptase (RT) activity. For construction of the cDNA expression library, cDNAs were synthesized from poly(A)-RNA of Jurkat cells with random hexamer primers using the SuperScript Choice system (Invitrogen Corp., Calsbad, CA) according to the manufacturer’s instructions. The synthesized cDNAs were inserted between the *Bst*XI sites of pMRX [27] using *Bst*XI adaptors (Invitrogen Corp., Carlsbad, CA), generating a retroviral cDNA expression library. For production of the retrovirus reporter vector, Plat-E cells were cotransfected with a retrovirus transfer plasmid: pMX-luc [30] and VSV-G using FuGENE 6 transfection reagent. Culture supernatant of Plat-E cells was collected 60 h after transfection and filtered through a 0.45 μm-pore syringe filter.

### Construction of expression vectors

POM121C (614–987) was amplified by PCR using total DNA isolated from surviving cell pools. The sequences of the primers used for amplification are shown in Table 1. The amplified cDNA was cloned into the pCR2.1-TOPO vector (Life Technologies Corp., Carlsbad, CA), and sequenced. The cDNA was then subcloned into the pMRX-HA-ires-puro vector [30]. Full-length POM121C (2–987) (GenBank accession number NM_001099415) was amplified by RT-PCR using total RNA isolated from HEK293 cells as the template. RT-PCR was performed with the PrimeScript II high-fidelity one-step RT-PCR kit (TaKaRa Bio., Inc., Shiga, Japan). The amplified RT-PCR products were inserted into pCR2.1 and sequenced. The cDNA was inserted into the *Bam*H I and *Not* I sites of pMRX-HA-ires-puro. To generate the POM121C-mutant expression constructs, the cDNAs of POM121C mutants were amplified by PCR using pMRX-HA-POM121C (614–987) as the template. The amplified PCR products were inserted into the pCR2.1-TOPO vector and sequenced. The cDNAs were inserted into the *Bam*H I and *Not* I sites of pMRX-HA-ires-puro. The resultant plasmids are referred to as pMRX-HA-POM121C (2–987), pMRX-HA-POM121C (614–987), pMRX-HA-POM121C (614–873), pMRX-HA-POM121C (686–987), pMRX-HA-POM121C (686–873), pMRX-HA-POM121C (801–987), pMRX-HA-POM121C (614–973) and pMRX-HA-POM121C (614–987: Δhelix). For construction of Strep-tag II fusion-GFP, -Cyclophilin A (CYPA) and -POM121C (614–987) protein expression vectors, the cDNAs of GFP and POM121C (614–987) were amplified by PCR using pMax-GFP (Lonza Japan Ltd., Tokyo, Japan), pcDNA-HA-CypA [31] or pMEX-HA-POM121C (614–987) as template. The amplified products were inserted into the *Kpn* I and *Xho* I sites of pCAG-One-STrEP-FLAG-tag (OSF) [32], and sequenced. The resultant plasmids are referred to as pCAG-OSF-GFP, pCAG-OSF-CypA and pCAG-OSF-POM121C (614–987). The *GST* gene was amplified by PCR using pGEX-4T-3 (GE Healthcare Life Sciences, Pittsburgh, PA) as the template. For generating expression vectors for GST-tagged proteins, a two-step PCR was carried out. The *GST* gene fragment and POM121C-mutant fragments were linked and amplified by fusion PCR. After cloning and confirmation of the nucleotide sequence, fused DNA fragments were excised by digestion with *Bgl* II and *Not* I and then inserted into the *Bam*H I and *Not* I sites of pMRX-HA-ires-puro. The resultant plasmids are referred to as pMRX-HA-GST, pMRX-HA-GST-POM121C (614–987), pMRX-HA-GST-POM121C (801–987), pMRX-HA-GST-POM121C (801–973) and pMRX-HA-GST-POM121C (801–987: Δhelix). pMX-luc was constructed by inserting an *Sma* I-*Hind* III DNA fragment carrying the *luciferase* gene of Igκcona-luc [33] into the *Hpa* I and *Hind* III sites of the pMX vector [34]. To generate the HIV-1_NL4-3_ vector carrying the firefly *luciferase* gene (pNL4-3luc-RC), fragments of the *env* gene and *luciferase* gene were fused by fusion PCR. The amplified fragments were extracted after digestion with *Hpa* I and *Not* I and inserted into the *Hpa* I and *Not* I sites of pNL-E [35], generating pNL4-3luc-RC. The coding sequence of HA-tagged POM121C (614–987) was amplified by PCR using pMRX-HA-POM121C (614–973) as the template. The PCR product was cloned into the pENTR/D-TOPO (Life Technologies Corp., Carlsbad, CA) vector. The coding sequence of HA-tagged POM121C (614–987) was transferred to the lentivirus destination vector CSII-EF-IB-RfA [36] using Gateway LR Clonase II Enzyme Mix (Life Technologies Corp., Carlsbad, CA). The resultant plasmid is referred to as pCSII-EF-IB-POM121C (614–987).

**Table 1:** List of primers used in this study.

Construction of expression vectors	forward primer	reverse primer
POM121C (2–987)	TTAGATCTGTGTGTAGCCCAGTGACTGT	AAGCGGCCGCCTACTTTTTGCGGGTGTGCT
POM121C (614–987)	TTAGATCTGGCTCCATATTCCAGTTTGG	AAGCGGCCGCCTACTTTTTGCGGGTGTGCT
POM121C (614–873)	TTAGATCTGGCTCCATATTCCAGTTTGG	AAGCGGCCGCCTACCCACTCTGTCCTGCTCCAA
POM121C (686–987)	TTAGATCTCCCTTTGGCTCAAGCGCCAA	AAGCGGCCGCCTACTTTTTGCGGGTGTGCT
POM121C (686–873)	TTAGATCTCCCTTTGGCTCAAGCGCCAA	AAGCGGCCGCCTACCCACTCTGTCCTGCTCCAA
POM121C (801–987)	TTAGATCTTTCTCCTTCGGTGCAGCCAC	AAGCGGCCGCCTACTTTTTGCGGGTGTGCT
POM121C (614–973)	TTAGATCTGGCTCCATATTCCAGTTTGG	AAGCGGCCGCCTAAGCCCCTGGGGTCTTGGATC
POM121C (614–987: Δhelix)	TTAGATCTGGCTCCATATTCCAGTTTGG	AAGCGGCCGCCTACTTTTTGCGGGTGTGCTGAGCCCCTGGGGTCTTGGATCCCGC
GFP	TTAGATCTGTGAGCAAGGGCGAGGAGCT	AAGCGGCCGCCTACTTGTACAGCTCGTCCATGC
OSF-CypA	GGGGTACCATGGTCAACCCCACCGT	CCGCTCGAGTTATTCGAGTTGTCCACAGT
OSF-POM121C (614–987)	TTTGGTACCGGCTCCATATTCCAGTTTGG	AAAACTCGAGCTACTTTTTGCGGGTGTGCT
GST	TTAGATCTTCCCCTATACTAGGTTATTG	AAGCGGCCGCCTATTTTGGAGGATGGTCGCCAC
GST-POM121C (614–987)	TTAGATCTTCCCCTATACTAGGTTATTG	AACTGGAATATGGAGCCTTTTGGAGGATGGTCGC
	GCGACCATCCTCCAAAAGGCTCCATATTCCAGTT	AAGCGGCCGCCTACTTTTTGCGGGTGTGCT
GST-POM121C (801–987)	TTAGATCTTCCCCTATACTAGGTTATTG	GCTGCACCGAAGGAGAATTTTGGAGGATGGTCGC
	GCGACCATCCTCCAAAATTCTCCTTCGGTGCAGC	AAGCGGCCGCCTACTTTTTGCGGGTGTGCT
GST-POM121C (801–973)	TTAGATCTTCCCCTATACTAGGTTATTG	AAGCGGCCGCCTAAGCCCCTGGGGTCTTGGATC
GST-POM121C (801–987: Δhelix)	TTAGATCTTCCCCTATACTAGGTTATTG	AAGCGGCCGCCTACTTTTTGCGGGTGTGCTGAGCCCCTGGGGTCTTGGATCCCGC
GST-POM121C (958–987)	TTAGATCTTCCCCTATACTAGGTTATTG	ATGGAAAATGAAGGGGCTTTTGGAGGATGGTCGC
	GCGACCATCCTCCAAAAGCCCCTTCATTTTCCAT	AAGCGGCCGCCTACTTTTTGCGGGTGTGCT
GST-POM121C (974–981)	TTAGATCTTCCCCTATACTAGGTTATTG	AAGCGGCCGCCTACTTTTTGCGGGTGTGCTGCCTTCGGGCCTGCAGTCGCTGTCGAGCTTTTGGAGGATGGTCGCCAC
pNL4-3luc-RC	TCTAGAATTCTCGAGTGTTAACTTGCTCAATGCCACAGCCATAGC	ACGCGGCCGCTTTACAATTTGGACTTTCCGCC
pENTR/D-TOPO-POM121C (614–987)	CACCACCATGAGGTCTTATCCA	CTACTTTTTGCGGGT

Sequences of primers used for PCR amplification in this study.

### Generation of cells stably expressing POM121C mutants

HEK293, HeLa, Jurkat and MT4C5 cells were transduced with retrovirus vectors for POM121C mutants and puromycin resistance and express POM121C-mutants. Cells stably expressing POM121C-mutants were obtained after selection with 4 μg/ml of puromycin for HEK293 and HeLa or 2 μg/ml of puromycin for Jurkat and MT4C5. PHA-stimulated PBMCs were transduced with lentivirus vectors for POM121C mutants and Blasticidin S resistance, and were cultured in the presence of 6 μg/ml of Blasticidin S.

### Infectivity assay

HEK293 and HeLa cells (3 × 10^5^) were infected for 24 h with 10 ng (p24) of VSV-G-pseudotyped NL4-3luc (VSV-G/NL4-3luc) or VSV-G-pseudotyped MLV-luc (VSV-G/MLV-luc) normalized by reverse transcriptase (RT) counts corresponding to 10 ng (p24) of VSV-G/NL4-3luc in 12-well plates. Cells were then harvested and lysed 24 h post-infection. MT4C5, Jurkat and PHA-PBMCs (5 × 10^5^) were infected for 24 h with 10 ng (p24) of VSV-G/NL4-3luc in 24-well plates, harvested and lysed 24 h post-infection. MT4C5 cells (2 × 10^5^) were infected for 72 h with 10 ng (p24) of NL4-3luc-RC in 24-well plates. Cells were then harvested and lysed 72 h post-infection. To determine luciferase activity, cell lysates were mixed with luciferase substrate and light emission was measured using a GloMax-Multi Detection System (Promega Corp., Madison, WI).

### Analysis of HIV-1 replication in human T cells

MT4C5 cells (1 × 10^5^) were exposed to HIV-1_NL4-3_ stock containing 10 pg of p24. Virus production was monitored for 8 days post-infection by measuring RT activity in the culture supernatants. Mean values from three independent experiments are shown.

### Western blotting

Whole-cell lysates were prepared as follows: cells were washed twice with phosphate-buffered saline (PBS) (-), suspended in PBS(-) (500 μl per 1 × 10^7^ cells) and mixed with an equal volume of 2 × sample buffer (4% sodium dodecyl sulfate, 125 mM Tris-HCl, pH 6.8, 10% 2-mercaptoethanol, 10% glycerol, and 0.002% bromphenol blue). Proteins were solubilized by heating for 5 min at 95°C. Samples were subjected to SDS-PAGE, transferred to PVDF membranes, and reacted with rat monoclonal antibody to HA (#11867423001, Sigma-Aldrich Co., St. Louis, MO), mouse monoclonal antibody to FLAG [29–22381, Wako Pure Chemical Industries, Ltd., Osaka, Japan], mouse monoclonal antibody to HIV-1 p24 (#ab9071, Abcam, Inc., Cambridge, MA), goat polyclonal antibody to HIV-1 gp120 (#ab21179, Abcam, Inc., Cambridge, MA), HIV-1-positive pooled serum from infected individuals (subtype B) [37], mouse monoclonal antibody to α-tubulin (#T6199, Sigma-Aldrich Co, St. Louis, MO), rabbit polyclonal antibody to CYPA (#BML-SA296, Enzo Life Sciences, Inc., Farmingdale, NY), or mouse monoclonal antibody to KPNB1 (#GTX22811, GeneTex, Inc., Irvine, CA). Membranes were then incubated with horseradish peroxidase-conjugated secondary antibody (#NA934 for anti-rabbit IgG, #NA931 for ant-mouse IgG, #NA933 for anti-human IgG, Amersham Biosciences, Piscataway, NJ), and proteins were visualized by Western Lightning Plus-ECL (PerkinElmer, Waltham, MA) or enhanced chemiluminescence (Pierce Biotechnology, Rockford, IL).

### Immunocytochemistry

HeLa cells (1 × 10^4^) were cultured for 48 h at 37°C in Nunc Lab-Tek II 8-well glass Chamber Slides (Thermo Fisher Scientific, Waltham, MA). Cells were then fixed with 4% paraformaldehyde for 10 min at room temperature followed by two washes in PBS(-). Fixed cells were permeabilized for 20 min at room temperature with 0.5% Triton X-100 in PBS(-) prior to incubation with antibodies. For staining, cells were incubated at 4°C overnight with primary antibodies diluted appropriately in 20% ImmunoBlock (DS Pharma Biomedical Co., Ltd., Osaka, Japan)/PBS(-). Cells were washed twice with PBS(-) and incubated with Alexa Fluor 594-conjugated secondary antibodies (Thermo Fisher Scientific, Waltham, MA) and Hoechst33342 (Thermo Fisher Scientific, Waltham, MA) for 2 h at room temperature. Samples were then washed twice with PBS(-) and mounted with VECTASHIELD (Vector Laboratories, inc., Burlingame, CA). Images were acquired by FV10i Confocal Laser Scanning Microscopy (OLYMPUS Corp., Tokyo, Japan)

### Luciferase reporter assay

HEK293, control vector-expressing HEK293 [22] or POM121C (614–987)-expressing HEK293 (POM121C [614–987]) cells (2 × 10^5^) in a 12-well plate were co-transfected with 0.45 μg of pNL4-3luc together with 0.05 μg of pGL4.84-EF1α-hRlucCP (Renilla-luc) [38] using FuGENE6 according to the manufacturer’s instructions. Cells were harvested and lysed 48 h post-transfection. The luciferase activity was measured using the GloMax-Multi Detection System (Promega Corp, Madison, WI).

### Quantification of viral cDNA

Measurement of reverse-transcribed viral cDNA in HIV-1-infected cells was performed as described previously [27]. Prior to infection, virus solution was treated with 100 U of RNase-free DNase I (Sigma-Aldrich Co, St. Louis, MO) in the presence of 10 mM MgCl_2_ for 30 min at 37°C. For infection, 7 × 10^5^ target EV-control HEK293 or POM121C (614–987)-expressing HEK293 cells were incubated for 12 or 24 h with VSV-G/NL4-3luc virus stock containing 23 ng of p24. Total cellular DNA was extracted using the DNeasy Blood & Tissue kit (Qiagen, Inc., Valencia, CA). VSV-G/NL4-3luc inactivated by incubation at 65°C for 30 min was used as a negative control. Primers U5-gag/F2 (5´-GTAGTGTGTGCCCGTCTGTTG-3´) (nt 553 to 573) and U5-gag/R2 (5´-CAAGCCGAGTCCTGCGT-3´) (nt 689 to 705) and probe U5-gag/probe2 (5´-FAM-TGGCGCCCGAACAGGGACTT-TAMRA-3´) (nt 636 to 655) were used for amplification and quantification of the *U5-gag* region of the HIV-1 late reverse transcription product. Primers 5’-CCCTCAGACC CTTTTAGTCAGTG-3’ (nt 9668–9690) and 5’-TGGTGTGTAGTTCTGCCAATCA-3’ (nt 77–98) and probe 5’-FAM-TGTGGATCTACCACACACAAGGCTACTTCC-TAMRA-3’ (nt 46–75) were used for amplification of the 2-LTR circle from the HIV-1_NL4-3_ cDNA. The integrated viral DNA was measured as described previously [39]. Briefly, the first PCR was carried out with an *Alu*-sequence-specific sense primer (*Alu*-HIV) and an antisense primer recognizing the LTR/*gag* region of the HIV sequence (M661). The first-round PCR samples were diluted with deionized distilled water by a factor of 10, and 5 μL was used as the template for the real-time PCR assay for measuring R/U5 DNA using M667, AA55 and HIV-FAM. For standardization, *β-globin* cDNA was quantified as described previously [40]. Real-time PCR was carried out with the StepOnePlus Real-Time PCR system (Applied Biosystems, Carlsbad, CA). The ratios of viral cDNA toβ-globin cDNA levels were calculated.

### Preparation of naked HIV-1 capsid core

Envelope-stripped HIV-1 virions (naked capsid cores) were prepared as described previously [41]. Briefly, HIV-1-containing culture supernatants were prepared by transiently transfecting HeLa cells with pNL4-3 using LipofectAMINE LTX PLUS (Invitrogen Corp., Carlsbad, CA). Two ml of 20% sucrose solution was placed at the bottom of SW55 centrifuge tubes and overlaid with 3 ml of the HIV-1-containing culture supernatant described above. Samples were then centrifuged for 60 min at 35,000 rpm at 4°C. Particulate HIV-1 was resuspended with PBS (-) containing a protease inhibitor cocktail (Nacalai Tesque, Inc., Kyoto, Japan). This suspension was loaded onto the top of a discontinuous sucrose density gradient composed of 1.0 ml 30% sucrose solution at the bottom of SW55 centrifuge tubes covered or not covered by 1.0 ml 0.1% Triton X-100 in 10% sucrose solution and then centrifuged in an SW55Ti rotor for 120 min at 35,000 rpm at 4°C. Particulate CA proteins were subjected to Western blotting with anti-HIV-1 env (gp120) or HIV-1-positive pooled serum from infected individuals (subtype B) to verify removal of HIV-1 env and MA proteins.

### Negative staining electron microscopy

Naked HIV-1 capsid cores isolated by ultracentrifugation were absorbed onto Formvar-coated copper grids, and stained with 2% phosphotungstic acid solution. The images were recorded with a Tecnai F20 transmission electron microscope (FEI company, Hillsboro, OR) at 200kV.

### Affinity precipitation of naked HIV-1 capsid cores with Strep-tag II fusion proteins

HEK293 cells were transfected with pCAG-OSF, pCAG-OSF-GFP, pCAG-OSF-POM121C (614–987) or pCAG-OSF-CypA, harvested 48 hr post-transfection and lysed in a 7 ml-Dounce homogenizer. Cell extracts were incubated with Strep-Tactin Sepharose for 2 h at 4°C. Purified Strep-tagged protein complexes were incubated with naked HIV-1 capsid cores for 2 h at 4°C. After extensive washing, Strep-tagged protein complexes were released by boiling in SDS-PAGE loading buffer and the proteins were analyzed by 12% SDS-PAGE by Western blotting using anti-FLAG or anti-p24 antibody.

### GST pull-down assays

Parental, EV-control or POM121C mutant-expressing HEK293 cells (7 × 10^7^) were harvested, resuspended in 1 ml of IP lysis buffer (25 mM Tris-HCl [pH 7.4], 150 mM NaCl, 1% NP40, 5% glycerol and protease inhibitor cocktail [Nacalai Tesque, Inc., Kyoto, Japan]) and incubated on ice for 30 min. The cell lysates were cleared by centrifugation at 8,000 × g for 3 minutes at 4°C. Part of the cell extract was reserved as input. Glutathione-Sepharose 4B (GE Healthcare Life Sciences, Pittsburgh, PA) was added to each cell extract and the mixtures were rotated for 2 h at 4°C. The beads were then washed five times with IP wash buffer (20 mM Tris [pH 7.4], 150 mM NaCl, 0.1% Triton X-100) and bound proteins were eluted with SDS sample buffer.

### TOF-mass spectrometry analysis to identify proteins interacting with POM121C mutants

Proteins were identified by liquid chromatography nano-electron spray ionization tandem mass spectrometry (LC-nESI-MS/MS) (QTRAP 5500 LC/MS/MS System, AB SCIEX, Concord, ON, Canada). Proteins excised from the gels were analyzed by LC-nESIMS/MS after separation on a HiQ sil C18W-3P column (0.1 mmΦ × 100 mm, KYA TECH Corporation, Tokyo, Japan). The proteins were eluted at a flow rate of 300 nL/min using water containing 0.1% (v/v) formic acid as the eluent A and acetonitrile containing 0.1% (v/v) formic acid as the eluent B with a linear gradient from 5% B to 45% B in 70 min. The data were analyzed using ProteinPilot^™^ software (Version 3, Applied Biosystems, Warrington, UK). Protein candidates were selected from those that had more than one distinct peptides with at least 95% confidence.

### Statistical analysis

**Fig 1:**
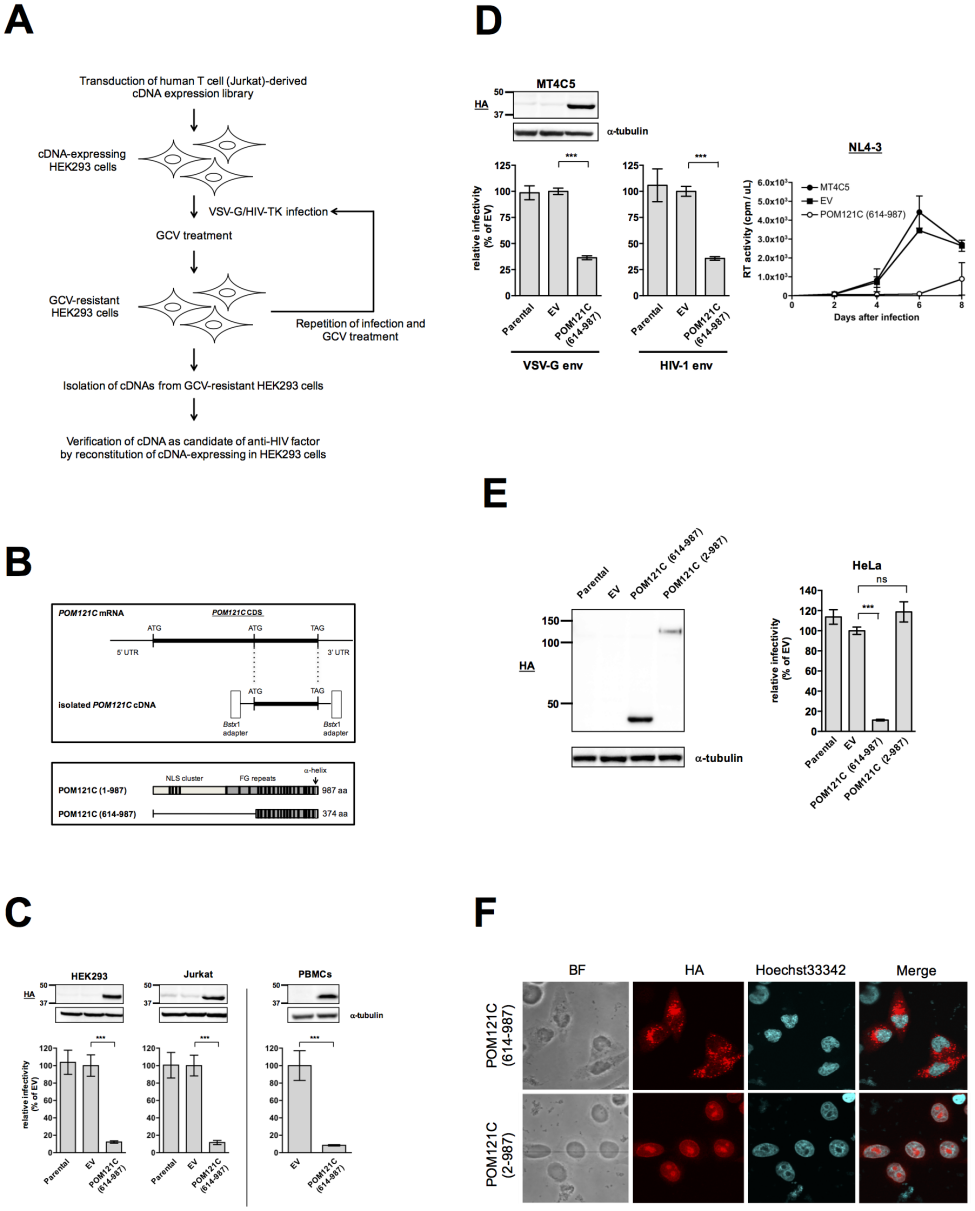
N-terminally truncated POM121C blocks HIV-1 infection in human cells. (A) HEK293 cells were transduced with a VSV-G-pseudotyped retroviral vector expressing a human T-cell-derived cDNA library. After several passages, the cells were infected with high titer HIV-1 capable of expressing HSV-TK (VSV-G/ NL4-3TK) and subjected to lethal selection with gancyclovir to eliminate infected cells. After repeated infection and selection, inserted cDNAs isolated from gancyclovir-resistant cells were amplified by PCR. The cDNAs were subcloned again into a retroviral vector and expressed in HEK293 cells to verify that they acted as anti-HIV factors. (B) Upper panel: Structures of *POM121C* mRNA and the isolated *POM121C* cDNA, which was linked to random hexamer primers and *Bst*XI adapters at both ends. Lower panel: schematic representation of the POM121C and POM121C (614–987) proteins. (C) Upper panels: Immunoblot analyses monitoring POM121C (614–987) expression in HEK293 (left panels), Jurkat (middle panels) or PHA-stimulated PBMCs (right panels). Lysates of parental cells (lane Parental), control vector-infected cells (lane EV), and cells stably expressing HA-tagged POMC121C (614–987) (lane POM121C [614–987]) were immunoblotted with anti-HA or anti-α-tubulin antibodies. Lower panels: Effects of POM121C (614–987) on viral infectivity. Parental, EV control and POM121C (614–987) cells were infected with VSV-G-pseudotyped NL4-3luc. Luciferase activity was measured 24 h after infection. Relative luciferase activities are shown as ratios (%) of the RLU of EV control cells with standard deviations calculated from at least three independent experiments. (D) Immunoblot analysis monitoring POMC121C (614–987) expression in MT4C5 cells stably expressing either EV or POM121C (614–987) (upper left panels). Parental, EV or POM121C (614–987) cells were infected with VSV-G-pseudotyped NL4-3luc (lower left panel) or replication-competent NL4-3luc-RC (lower middle panel). Relative luciferase activities are shown as ratios (%) of the RLU of EV control MT4C5 cells with standard deviations calculated from three independent experiments. MT4C5, EV or POM121C (614–987) cells were infected with the HIV-1_NL4-3_ strain (lower right panel). Viral production was monitored by measuring RT activity in the culture supernatants. Mean values and standard deviations in three independent experiments are shown. (E) Left panel: Immunoblot analysis monitoring HA-tagged POMC121C (614–987) expression in parental HeLa cells and those transduced with EV, POM121C (614–987) or POM121C (2–987) expression vectors. Right panel: Effects of POM121C (614–987) on VSV-G-pseudotyped HIV-1 infectivity in HeLa cells. Parental, EV, POM121C (614–987) or POM121C (2–987) HeLa cells were infected with VSV-G-pseudotyped NL4-3luc. Luciferase activity was measured 24 h after infection. Relative luciferase activities are shown as ratios (%) of the RLU of EV control cells with standard deviations calculated from three independent experiments. (F) Localization of POM121C (614–987) or POM121C (2–987) in HeLa cells stably expressing each of the proteins. Cells were multistained with anti-HA (red) to detect HA-tagged proteins and Hoechst33342 (blue) as nuclear counterstaining. Statistical significance was determined by unpaired two-tailed Student’s *t* test (C: panel PBMCs) or one-way analysis of variance (ANOVA) with Dunnett’s multiple comparison test (C: panels HEK293 and Jurkat, D, and E). ns, not significant (*P*>0.05); ***P<0.001.

All data were obtained from at least three independent experiments. The average values are presented with error bars indicating the standard deviation (SD) and the statistical significance was analyzed using one-way analysis of variance (ANOVA) with Dunnett’s multiple comparison test, or Student’s *t*-test. All the statistical analyses were performed using Prism 6 software (GraphPad Software, Inc). *P* values below 0.05 (P<0.05, *; P<0.01, **; P<0.001,***) were considered significant. Unpaired two-tailed Student’s *t*-test was used for the data shown in Figs 1C (panel PBMCs) and 7C to test whether the means of the two groups were significantly different. One-way analysis of variance (ANOVA) with Dunnett’s multiple comparison test was used for the data shown in Figs 1C (panels HEK293 and Jurkat), 1D, 1E, 2A, 3B, 3D, 4C, 4E, 5B, 5C, 6D, 6G and 7A to determine whether the means of multiple groups were significantly different from a single group.

**Fig 2:**
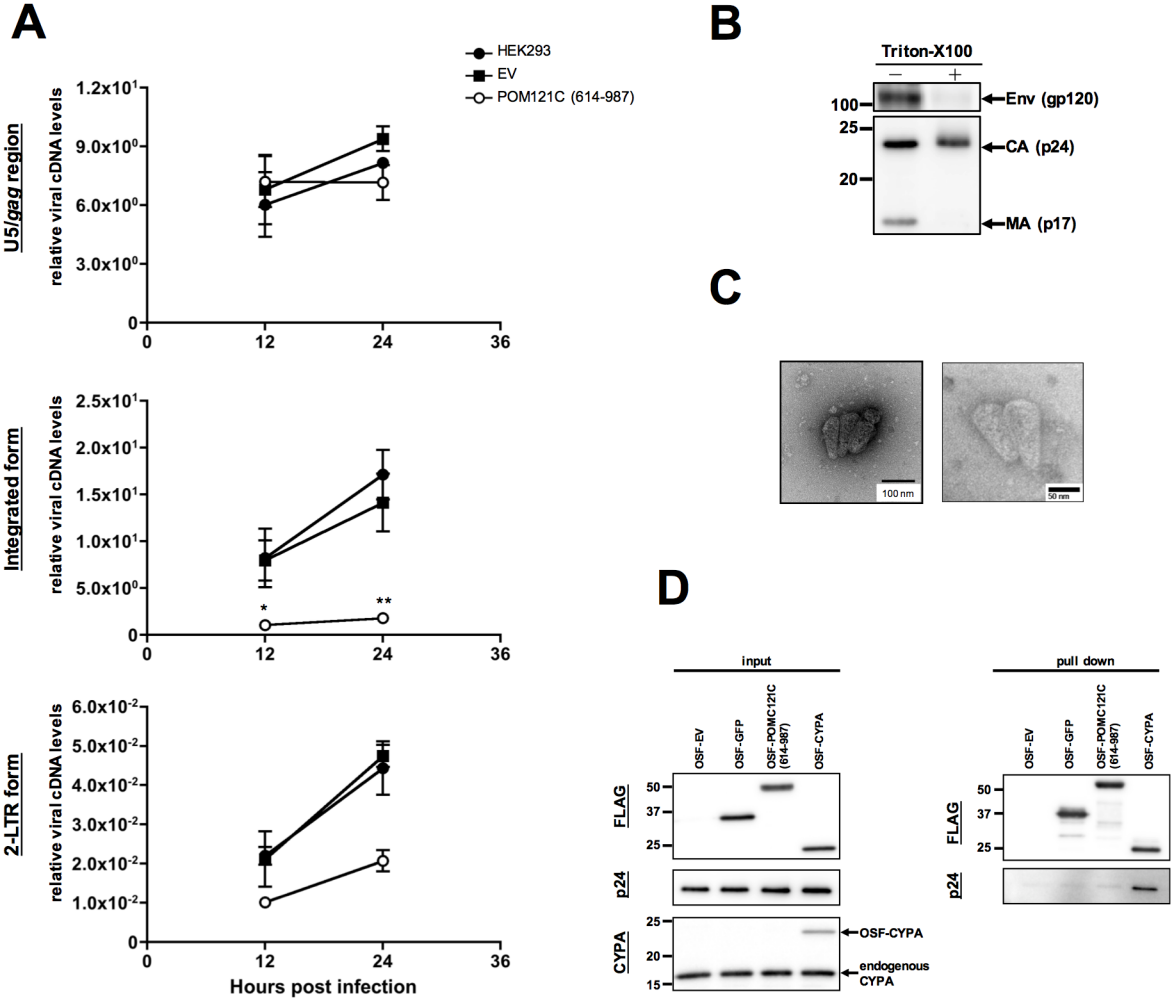
POM121C (614–987) inhibits HIV-1 replication after reverse transcription. (A) Quantitative DNA-PCR analysis of viral cDNA metabolism after VSV-G/NL4-3luc infection of HEK293-derived cells. Total DNA was extracted at the indicated times (12 and 24 h) and analyzed for the amount of viral cDNAs with a primer set recognizing the *U5-gag* region (top panel), integrated form (middle panel) or 2-LTR form (bottom panel). In cells exposed to heat-inactivated virus, the amount of viral DNA was below the level of detection in the real-time PCR assay. Mean values and standard deviations in three independent experiments are shown. (B) Immunoblot analysis of HIV-1 virions before or after stripping the envelope. Concentrated virions were subjected to step-gradient centrifugation in the absence (-) or presence (+) of 0.1% of Triton-X100. The pellets were immunoblotted using anti-HIV-1 env (gp120) or HIV-1-positive pooled serum from infected individuals (subtype B). (C) Electron micrographs showing envelope-stripped virions of HIV-1. TEM images of negatively stained naked HIV-1 cores prepared from HIV-1_NL4-3_ virions (left panel, lower magnification; right panel, higher magnification). Representative fields are shown. Bar scales indicate 100 nm (left panel) and 50 nm (right panel). (D) Immunoblot analyses showing OSF-tagged proteins bound to naked HIV-1 cores. Input naked HIV-1 cores and purified Strep-tagged proteins were immunoblotted using anti-FLAG (input, top panel), anti-p24 (input, middle panel), or anti-CYPA used as an internal control (input, bottom panel). Bound protein complexes were pulled-down and analyzed by immunoblotting with anti-FLAG or anti-p24 antibodies (right panels). Statistical significance was determined by one-way analysis of variance (ANOVA) with Dunnett’s multiple comparison test (A: middle panel). *P<0.05, **P<0.01.

**Fig 3:**
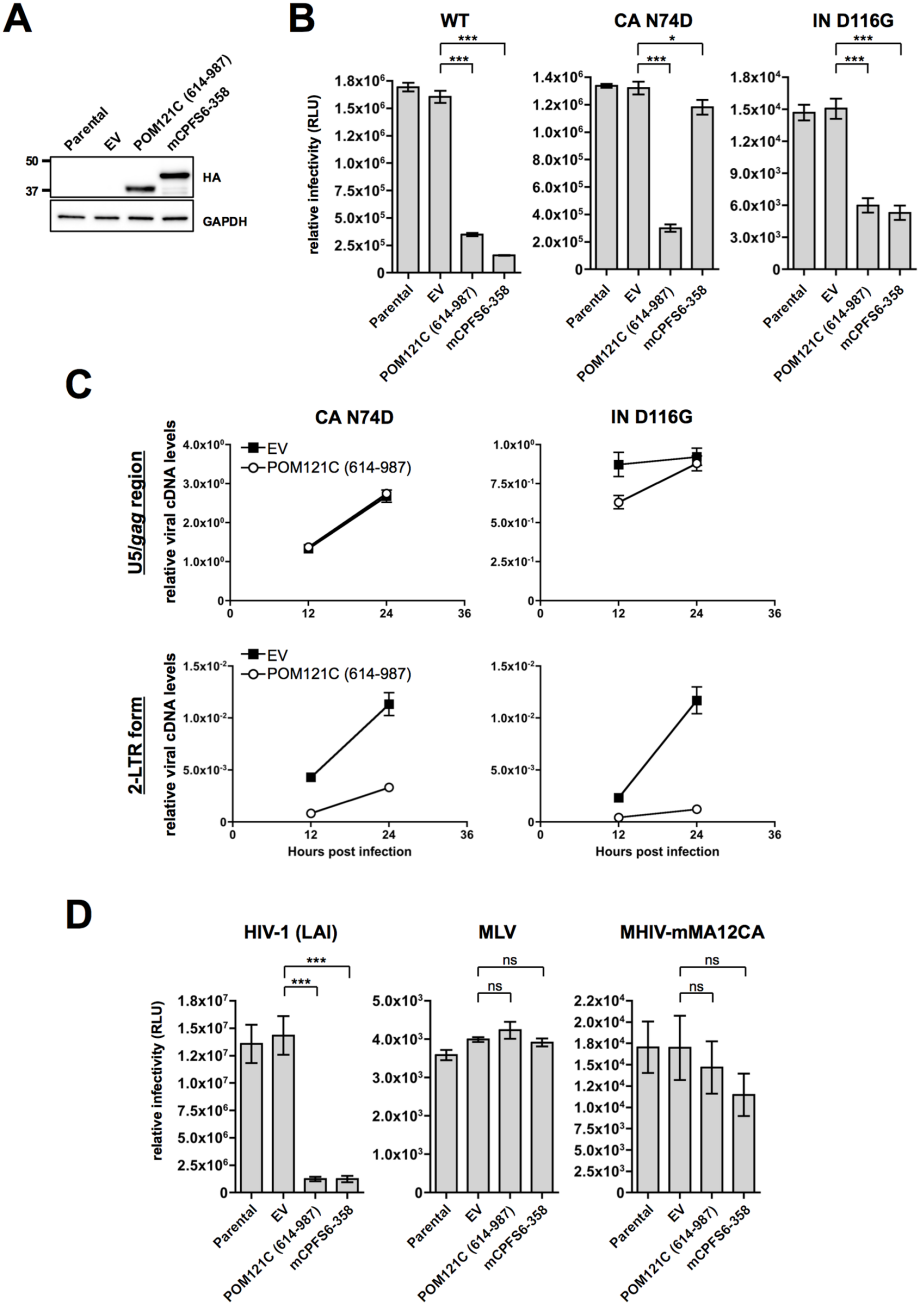
POM121C (614–987) inhibits nuclear import and integration in a Gag-dependent manner. (A) Immunoblot analysis of HEK293 cells expressing POM121C (614–987) or mCPSF6-358HA. Whole-cell lysates were immunoblotted using antibodies against HA or GAPDH (used as an internal control). (B) Effects of POM121C (614–987) or mCPSF6-358 on infection with HIV-1. Parental, EV, POM121C (614–987) or mCPSF6-358 cells were infected with VSV-G/NL4-3-luc (WT), VSV-G/NL4-3-luc CA N74D (CA N74D) or VSV-G/NL4-3-luc IN D116G (IN D116G). Luciferase activity was measured 24 h after infection. Relative luciferase activities are shown with standard deviations calculated from three independent experiments. (C) Relative levels of viral cDNA synthesized after VSV-G/NL4-3 CA N74D-luc or VSV-G/NL4-3 IN D116G-luc infection. Total DNA was isolated from a portion of the cells at the indicated times after infection. Experiments were done as in Fig 2A. Mean values and standard deviations in three independent experiments are shown. (D) Effects of POM121C (614–987) or mCPSF6-358 on infection with HIV-1, MLV or HIV/MLV chimeric virus, MHIV-mMA12CA. Parental, EV, POM121C (614–987) or mCPSF6-358 cells were infected with VSV-G/LAI-luc [HIV-1 (LAI)], VSV-G/MLV-luc (MLV) or VSV-G/MHIV-mMA12CA-luc (MHIV-mMA12CA). Luciferase activity was measured 48 h after infection. Relative luciferase activities are shown with standard deviations calculated from three independent experiments. Statistical significance was determined by one-way analysis of variance (ANOVA) with Dunnett’s multiple comparison test (B and D). ns, not significant (*P*>0.05); *P<0.05, ***P<0.001.

**Fig 4:**
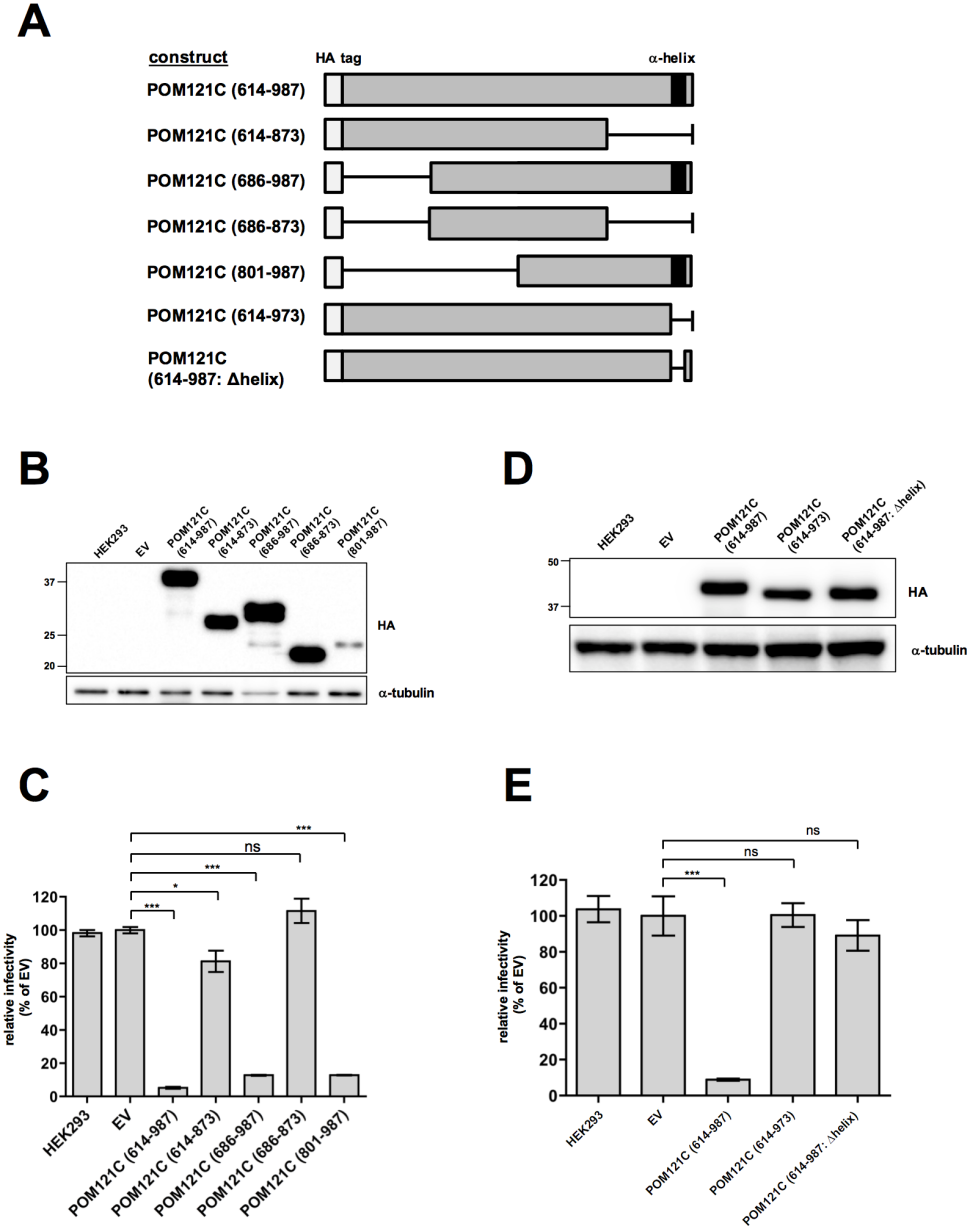
The C-terminal α-helix structure is required for HIV-1 inhibition. (A) Schematic presentation of POM121C mutants. The black boxes indicate the domain of a prospective helical-structure. The white boxes indicate HA-epitope tag. (B and D) Immunoblot analysis of HEK293 cells expressing truncated forms of POM121C. Whole-cell lysates were immunoblotted using anti-HA or anti-α-tubulin antibodies. (C and E) Effects of POM121C mutants on viral infectivity. HEK293 cells stably expressing the indicated POM121C mutants were infected with VSV-G/NL4-3luc. Luciferase activity was measured 24 h after infection. Relative luciferase activities are shown as ratios (%) of the RLU of EV control cells with standard deviations calculated from three independent experiments. Statistical significance was determined by one-way analysis of variance (ANOVA) with Dunnett’s multiple comparison test (C and E). ns, not significant (*P*>0.05); *P<0.05, ***P<0.001.

**Fig 5:**
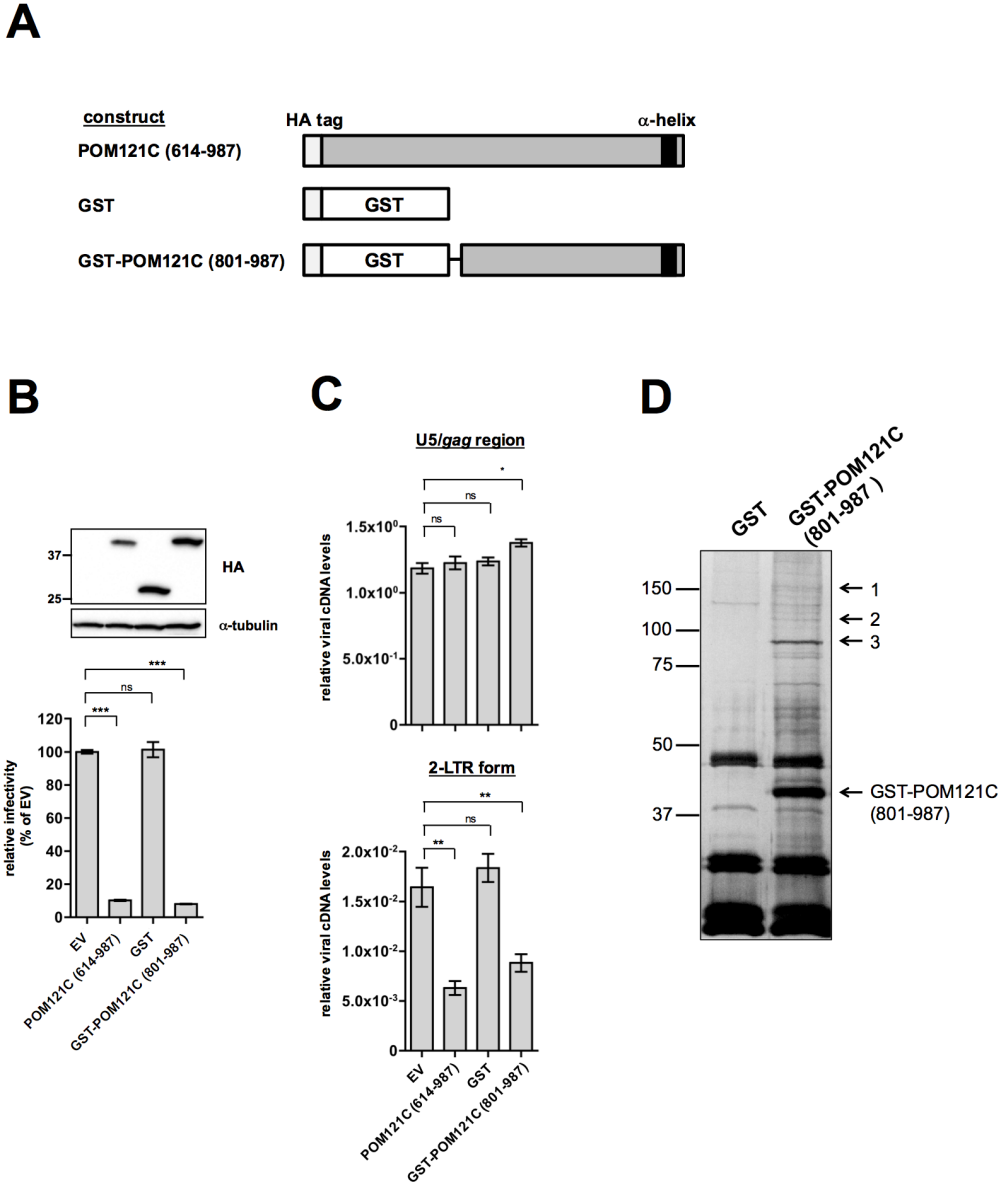
GST-fused POM121C (801–987) inhibits HIV-1 infection. (A) Schematic presentation of HA-tagged GST-POM121C (801–987). (B) Immunoblot analysis of HEK293 cells expressing HA-tagged GST or GST-POM121C (801–987) (upper panel). Whole-cell lysates were immunoblotted using anti-HA or anti-**α**-tubulin antibodies. Effects of GST-POM121C (801–987) on viral infectivity (lower panel). HEK293 cells stably expressing HA-tagged GST, POM121C (614–987) or GST-POM121C (801–987) were infected with VSV-G/NL4-3luc. Luciferase activity was measured 24 h after infection. The mean luciferase value from EV control cells was arbitrarily set as 100% and standard deviations in three independent experiments are shown. (C) Relative amounts of viral cDNA synthesized after VSV-G/NL4-3luc infection. Total DNA was isolated from the cells 12 h after infection. Viral cDNA synthesis was quantified by real-time PCR with a primer set recognizing the *U5/gag* region (upper panel) or 2-LTR form (lower panel) as described in EXPERIMENTAL PROCEDURES. In cells exposed to heat-inactivated virus, viral DNA was below the level of detection by real-time PCR assay. Mean values and standard deviations in three independent experiments are shown. (D) Purified GST or GST- POM121C (801–987) complexes were subjected to SDS-PAGE and processed for Silver Staining. The arrows indicate three different gel portions analyzed by mass spectrometry. Statistical significance was determined by one-way analysis of variance (ANOVA) with Dunnett’s multiple comparison test (B: lower panel, and C). ns, not significant (*P*>0.05); *P<0.05, **P<0.01, ***P<0.001.

**Fig 6:**
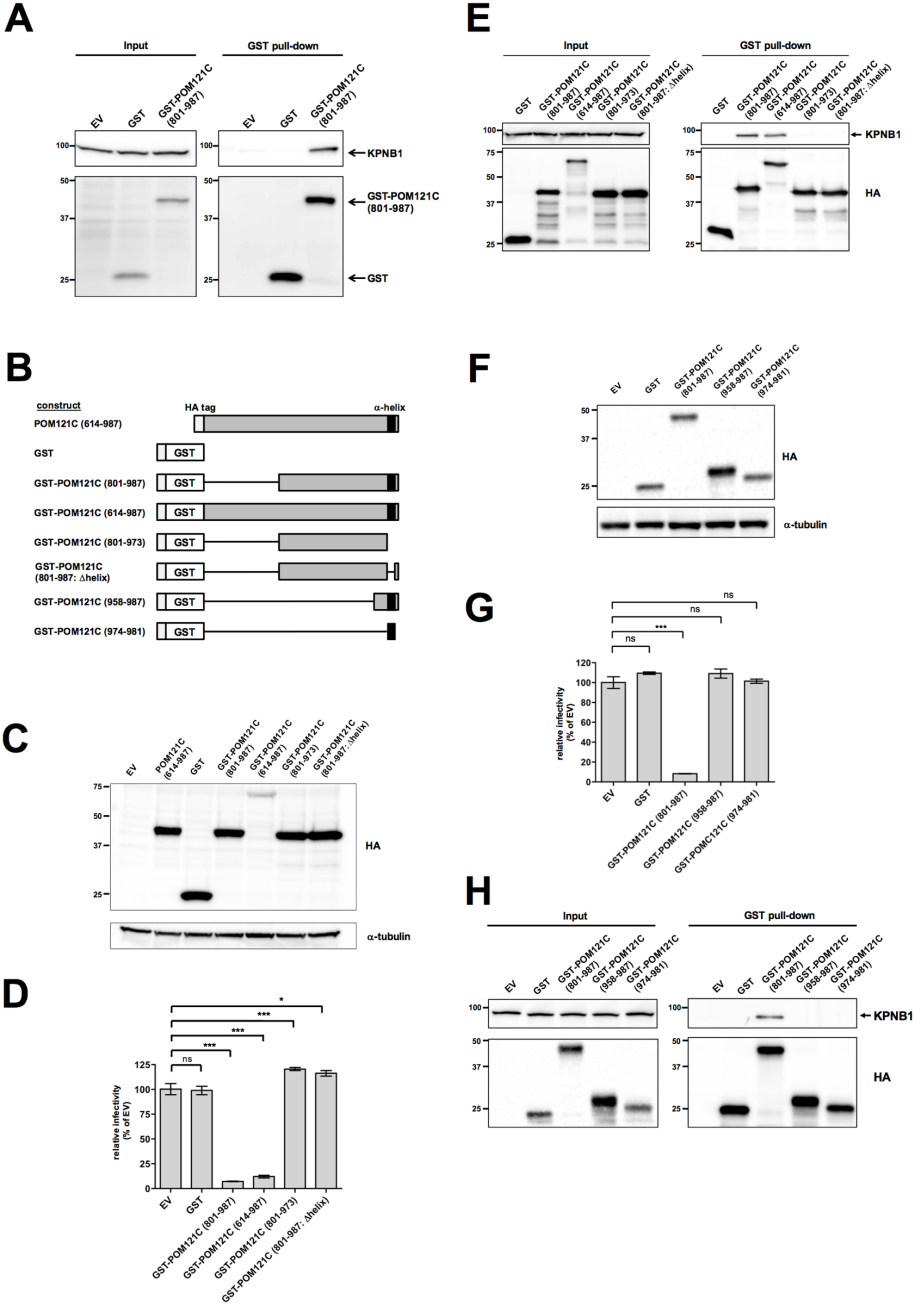
POM121C mutant binding to KPNB1 correlates with inhibition of HIV-1 infection. (A) Immunoblot analysis showing GST-POM121C (801–987) bound to KPNB1 in HEK293 cells. Cell lysates were analyzed by immunoblotting either directly (left panels: input) or following GST pull-down experiments. Purified GST or GST-POM121C (801–987) complexes were immunoblotted using anti-GST or anti-KPNB1 antibodies. (B) Schematic presentation of POM121C mutants fused with HA-tagged GST. (C) Immunoblot analysis of HEK293 cells expressing truncated forms of POM121C mutants fused with HA-tagged GST. Whole-cell lysates were immunoblotted using anti-HA or anti-**α**-tubulin antibodies. (D) Effect of GST-POM121C mutants on viral infectivity. HEK293 cells stably expressing GST or the indicated GST-POM121C mutants were infected with VSV-G/NL4-3luc. Luciferase activity was measured 24 h after infection. Relative luciferase activities are shown as ratios (%) of the RLU of EV control cells with standard deviations calculated from three independent experiments. (E) Cell lysates were analyzed by immunoblotting with anti-HA and anti-KPNB1 antibodies either directly (left panels: input) or following GST pull-down experiments (right panels: GST pull-down). (F) Immunoblot analysis of HEK293 cells expressing truncated forms of GST-POM121C mutants. Experiments were done as described in (C). (G) Effects of the indicated GST-POM121C mutants on viral infectivity. Experiments were done as described in (D). Relative luciferase activities are shown as ratios (%) of the RLU of EV control cells with standard deviations calculated from three independent experiments. (H) GST pull-down analysis showing GST-POM121C (801–987) bound to intracellular KPNB1. Cell lysates were analyzed by immunoblotting either directly (left panels: Input) or subsequent to GST pull-down (right panels) with anti-HA (lower panels) or anti-KPNB1 (upper panels) antibodies. Statistical significance was determined by one-way analysis of variance (ANOVA) with Dunnett’s multiple comparison test (D and G). ns, not significant (*P*>0.05); *P<0.05, **P<0.01, ***P<0.001.

**Fig 7:**
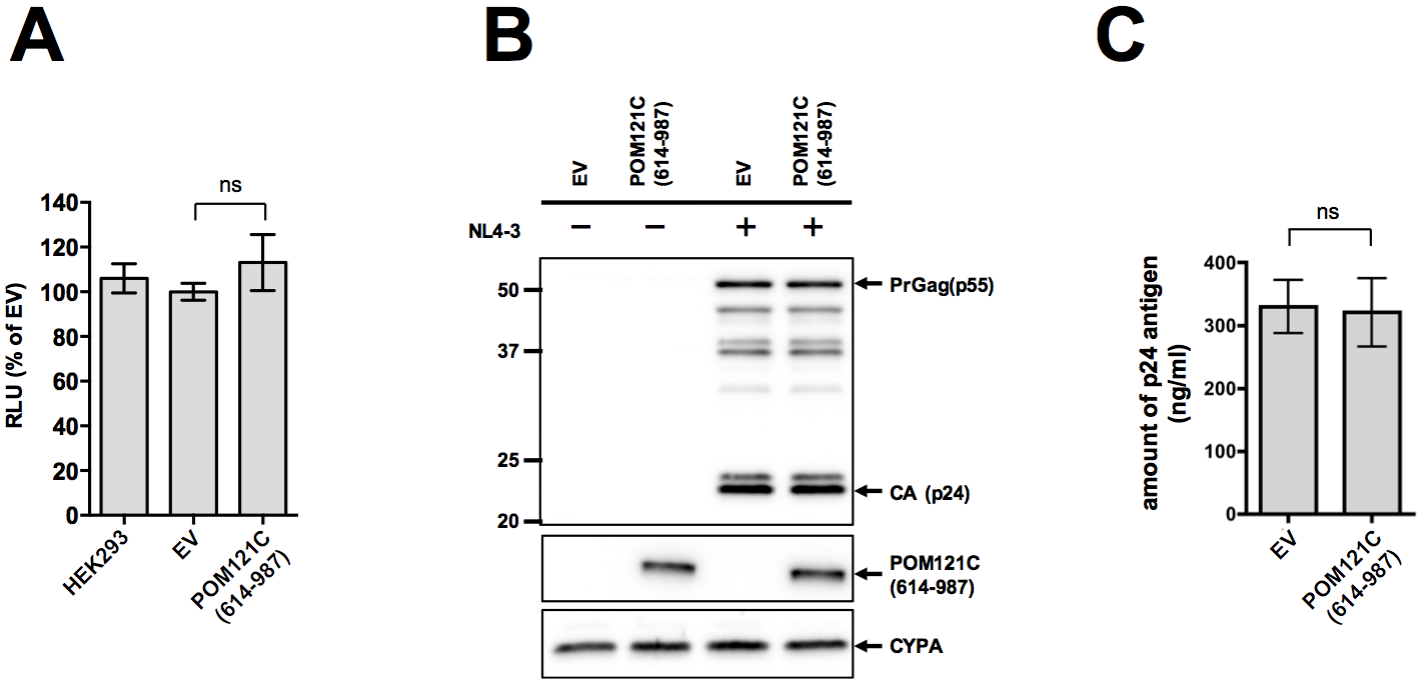
POM121C (614–987) does not affect the late phase of HIV-1 replication. (A) Effects of POM121C (614–987) on HIV-1 infection at the late stages. HEK293, EV or POM121C (614–987) cells were co-transfected with pNL4-3luc together with pGL4.84-EF1**α**-hRlucCP (Renilla-luc). Two days after transfection, luciferase activity was determined by the Dual-Luciferase assay system. The RLU of each firefly luciferase activity relative to renilla luciferase activity is given. The results are shown as an average of three independent experiments with standard deviations. (B) Immunoblot analysis of viral proteins in HEK293-derived cells transiently transfected with the proviral plasmid, pNL4-3. EV or POM121C (614–987) cells were transfected with 1.0 μg of pNL4-3. The cells were harvested 48 h post-transfection and lysates were subjected to immnoblot analyses with anti-HIV-1 p24 (top panel; the upper arrow indicated precursor Gag [PrGag], the lower arrow indicated CA), anti-HA (middle panel) and anti-CYPA (bottom panel). One representative set of results from three independent experiments is shown. (C) The amounts of p24 antigen in the supernatants of transfected cells were quantified with HIV-1 CA (p24) ELISA. Mean values and standard deviations are shown from three independent experiments. Statistical significance was determined by one-way analysis of variance (ANOVA) with Dunnett’s multiple comparison test (A), or unpaired two-tailed Student’s *t* test (C). ns, not significant (*P*>0.05).

## Results

### N-terminally truncated POM121C blocks HIV-1 infection

To identify proteins that interfere with HIV-1 infection, we employed a functional screen using a cDNA expression library. The details of the method of screening were essentially the same as those previously reported [27] except that complementary DNAs were synthesized from poly(A)-RNA of the Jurkat human T-cell line with random hexamer primers. In brief, HEK293 cells transduced with a cDNA expression library in a murine leukemia virus (MLV) vector were challenged with vesicular stomatitis virus glycoprotein (VSV-G)-pseudotyped recombinant HIV-1 capable of expressing herpes simplex virus thymidine kinase (HIV-1-TK) [27] followed by selection of uninfected cells in the presence of ganciclovir (GCV). After several rounds of HIV-1-TK infection and subsequent GCV selection, we characterized surviving cell clones resistant to HIV-1 infection and obtained a cDNA that encodes an N-terminally truncated form of POM121C (POM121C [614–987]) (Fig 1A and 1B). Stable expression of POM121C (614–987) in HEK293 cells with an influenza virus hemagglutinin (HA) tag, resulted in potent suppression of single-round infection of these cells with VSVG-pseudotyped NL4-3luc reporter virus (Fig 1C, left panel). Similar inhibition of HIV-1 infection was observed in the Jurkat cell line stably expressing POM121C (614–987) (Fig 1C, middle panel). Transduction of phytohemagglutinin/interleukin-2-stimulated peripheral blood mononuclear cells (PBMCs) with a lentivirus vector expressing HA-tagged POM121C (614–987) also resulted in a marked resistance to VSV-G-pseudotyped NL4-3luc infection (Fig 1C, right panel). Stable expression of POM121C (614–987) in MT4C5 cells markedly reduced the infectivity of VSV-G-pseudotyped HIV-1 (Fig 1D, left panel), replication-competent HIV-1 reporter virus, NL4-3luc-RC (Fig 1D, middle panel) and NL4-3 in spreading infection assays (Fig 1D, right panel). Neither the surface expression of CD4 and CXCR4 nor cell growth was affected by stable POM121C (614–987) expression (data not shown). However, stable expression of HA-tagged full-length POM121C in HeLa cells did not significantly influence VSV-G-pseudotyped HIV-1 infection (Fig 1E, right panel). A weaker expression of full-length POM121C (Fig 1E, left panel) may partly explain this lack of inhibition, but immuno-fluorescence studies revealed that the POM121C (614–987) was localized predominantly in the cytoplasm of HeLa cells, whereas full-length POM121C was found in the nucleus (Fig 1F). These results suggest that POM121C (614–987) expressed in the cytoplasm reduced the infectivity of HIV-1 in human cells.

### POM121C (614–987) inhibits HIV-1 replication in the early stage of HIV-1 infection

Having shown that POM121C (614–987) restricts single-round and spreading HIV-1 infection, we next addressed which stage of HIV-1 replication is targeted by this protein. Viral cDNA synthesis was assessed by quantitative polymerase chain reaction (qPCR) studies in HEK293 cells expressing or not expressing POM121C (614–987) (Fig 2A). Following infection with VSV-G-pseudotyped NL4-3luc, the late reverse transcription (RT) product (Fig 2A, top panel) in cells expressing POM121C (614–987) was produced similarly to control cells up to 24 hr after infection. Notably, expression of POM121C (614–987) resulted in a marked decrease of the amount of integrated provirus, consistent with the results obtained with reporter virus infection (Fig 2A, middle panel). POM121C (614–987) also moderately reduced production of the 2-LTR form that accumulates exclusively in the nucleus and is therefore recognized as a marker of nuclear import of viral cDNA (Fig 2A, bottom panel).

The moderate and marked reduction in the 2-LTR and integrated provirus forms, respectively, in the presence of POM121C (614–987) implies possible blockade by POM121C (614–987) at the nuclear import, nuclear trafficking, or integration stage. Recent studies indicate that HIV-1 nuclear entry is regulated by viral capsid (CA) [14, 42]. To investigate potential interactions between POMC121C (614–987) and the viral core in HIV-1-infected cells, we performed HIV-1 CA core binding assays with envelope-stripped HIV-1 virions. The integrity of the prepared naked capsid core was verified biochemically by Western blotting (Fig 2B) showing loss of the envelope and matrix proteins after stripping, and confirmed morphologically by electron microscopy (Fig 2C) showing the cone-shaped viral core. Pull-down assays with naked HIV-1 cores and purified OSF-tagged proteins revealed that POM121C (614–987) did not interact significantly with the capsid core (Fig 2D, panel pull down). In contrast, the association of the HIV-1 core with Cyclophilin A (CYPA), known to be an HIV-1 core-binding protein [43], was clearly demonstrated (Fig 2D, panel pull down). These results indicate that POM121C (614–987) inhibits HIV-1 infection after reverse transcription without binding to HIV-1 core.

### POM121C (614–987) inhibits nuclear import and integration

To gain more insight into blockade of infectivity after completion of RT, HEK293 cells expressing POM121C (614–987) or mCPSF6-358, which had been reported to block nuclear import of viral cDNA [15], were infected with VSV-G-pseudotyped NL4-3luc carrying the N74D mutation in the CA or the D116G mutation in the integrase (IN). The N74D mutation significantly reduces the binding affinity of mCPSF6-358 to HIV-1 CA, resulting in viral escape from restriction by mCPSF6-358 [15], while the D116G mutation disables HIV-1 for integration within host chromatin without affecting RT or nuclear entry [28]. We stably expressed POM121C (614–987) or mCPSF6-358 in HEK293 cells for single-round reporter virus infection assays. Expression of POM121C (614–987) or mCPSF6-358 was verified by Western blotting (Fig 3A). As shown in Fig 3B, mCPSF6-358 weakly affected N74D HIV-1 infection, consistent with a previous report [15]. POM121C (614–987) potently or moderately inhibited infection with N74D or D116G mutant viruses, respectively (Fig 3B, panels CA N74D and IN D116G). Kinetic studies of viral cDNAs following infection of cells expressing POM121C (614–987) with the N74D or D116G viruses showed that the late RT form was produced at levels similar to those of control cells (Fig 3C, upper panels), while accumulation of the 2-LTR form was decreased (Fig 3C, lower panels). This suggests that the action of POM121C (614–987) does not depend on the route of nuclear import of HIV-1 cDNA. The moderate inhibition of D116G virus infection by POM121C (614–987) suggests that POM121C (614–987) not only blocks nuclear import but also affects integration of HIV-1 providing that the D116G virus lacks the integrase activity. In this case, the overall inhibitory effects of POM121C (614–987) on this mutant virus may be proportionally alleviated. Additionally, overexpression of POM121C (614–987) did not inhibit the infectivity of the VSV-G-pseudotyped murine leukemia virus (MLV)-based vector, suggesting that POM121C (614–987) expressed in the cytoplasm has no effect on gamma retrovirus infection (Fig 3D, middle panel). A previous study had shown that CA is a pivotal determinant of retrovirus infection in non-dividing cells, using an HIV-1-based chimeric virus MHIV-mMA12CA, in which the entire Matrix (MA) and CA proteins were replaced by those of MLV [14]. Having shown that POM121C (614–987) does not inhibit MLV infection in dividing cells, we next investigated whether it inhibits infection with MHIV-mMA12CA, which should not encounter inhibition of nuclear entry in dividing cells. Indeed, POM121C (614–987) did not significantly inhibit MHIV-mMA12CA infection in dividing cells (Fig 3D, right panel). These results indicate that Gag is a genetic determinant of POM121C (614–987)-mediated HIV-1 restriction although POM121C (614–987) did not seem to interact with the capsid core (Fig 2D).

### Prevention of HIV-1 infection by POM121C (614–987) requires the C-terminal α-helix structure

POM121C (614–987) contains FG repeat motifs like other nucleoporins and a putative short α–helix motif at the extreme C-terminus. To determine the functional importance of these motifs, a series of truncation mutants was prepared and tested for their anti-HIV-1 activity in HEK293 cells (Fig 4A). Single-round infection studies revealed that mutants containing the α-helix structure (POM121C [686–987] and POM121C [801–987]) efficiently inhibited HIV-1 infection, although POM121C (801–987) was poorly expressed (Fig 4B and 4C). To assess the contribution of the C-terminal α-helix structure in POM121C (614–987) to the prevention of HIV-1 infection, we tested mutants with a small truncation or deletion of the α-helix motif of POM121C (614–987), POM121C (614–973) and POM121C (614–987: Δhelix). The deletion of the α–helix motif did not essentially alter subcellular localization (data not shown). Both α-helix motif mutants were expressed at similar levels to POM121C (614–987), but had lost inhibitory activity (Fig 4D and 4E). These results indicate that the α-helix motif plays an essential role in POM121C (614–987)-mediated HIV-1 restriction.

We next sought to determine how POM121C (614–987) interferes with HIV-1 infection. Because we failed to co-immunoprecipitate any viral protein with POM121C (614–987), a mammalian expression vector for POM121C (801–987) fused to the GST protein (GST-POM121C [801–987]) was constructed (Fig 5A and 5B). This fusion protein efficiently inhibited HIV-1 infection in HEK293 cells (Fig 5B, lower panel), did not affect the synthesis of the late RT product and reduced the amount of the 2-LTR form of viral cDNA in the same manner as POM121C (614–987) (Fig 5C). GST pull-down experiments using HEK293 cells stably expressing GST-POM121C (801–987) reproducibly demonstrated that several proteins specifically co-precipitated with GST-POM121C (801–987) (Fig 5D). Analyses of the isolated protein bands by mass spectrometry revealed that the most prominent, band 3, contains the karyopherin, importin subunit beta-1 (KPNB1) (Table 2).

**Table 2:** Cellular proteins bound to GST-POM121C (801–987) identified by mass spectrometry.

Band no.	Number of matching peptides (95%)	Accession number	Protein name
1	3	Q4VCS5	Angiomotin
1	2	O43795	Myosin-Ib
1	3	Q6WCQ1	Myosin phosphatase Rho-interacting protein
2	5	O94832	Myosin-Id
2	6	Q9UHB6	LIM domain and actin-binding protein 1
2	6	P57740	Nuclear pore complex protein Nup107
3	7	Q14974	Importin subunit beta-1 (KPNB1)
3	4	Q8N1F7	Nuclear pore complex protein Nup93
3	2	P49589	Cysteinyl-tRNA synthetase, cytoplasmic

Cellular proteins found by mass spectrometry to associate with GST-POM121C (801–987). Band no. refers to the arrow numbers on the right side of Fig 5D.

### POM121C (801–987) binding to KPNB1 correlates with inhibition of HIV-1 infection

Immunoblotting studies revealed that KPNB1 specifically interacts with GST-POM121C (801–987) (Fig 6A, panel GST pull-down). To investigate the functional consequences of POM121C (801–987) binding to KPNB1, truncation or deletion mutants of GST-POM121C (614–987) were stably expressed in HEK293 cells (Fig 6B and 6C) and tested for HIV-1 inhibition and KPNB1 binding (Fig 6D and 6E). As expected from the mutational studies of POM121C (614–987), two GST fusion proteins lacking the C-terminal α-helix motif were unable to inhibit HIV-1 infection, whereas those containing the C-terminal part of the FG repeat motifs and the α-helix motif did block HIV-1 in the single-round infection assay (Fig 6D). The binding of these mutants to KPNB1 correlated with the inhibition of HIV-1 infection (Fig 6E, panel GST pull-down). GST proteins linked to the extreme C-terminus of POM121C containing the α-helix motif but not the FG repeat motifs or simply to the α-helix motif failed to inhibit HIV-1 infection or to interact with KPNB1 (Fig 6F, 6G and 6H), suggesting that the α-helix motif alone is not sufficient for HIV-1 blockade or binding to KPNB1. These results suggest that POM121C (614–987)-mediated HIV-1 inhibition is closely linked to its binding to KPNB1.

### POM121C (614–987) does not affect the late phase of HIV-1 replication

Transient transfection with the proviral plasmid pNL4-3luc allows for bypassing the replication processes up to the integration stage and mimics the expression of viral genes from the integrated HIV-1 provirus. EV-control- or POM121C (614–987)-expressing HEK293 cells were transfected with the NL4-3luc provirus plasmid which has the *luciferase* gene in the *nef* position. This resulted in similar levels of reporter gene activity and newly-synthesized viral protein (Fig 7A and 7B). Likewise, POM121C (614–987) did not affect the release of HIV-1 CA protein from cells transfected with the pNL4-3 provirus plasmid (Fig 7C). These results suggest that POM121C (814–987) does not inhibit the late phase of the HIV-1 replicative cycle.

## Discussion

Based on similar late RT product synthesis 24 h post infection, we have shown here that POM121C (614–987) does not bind to the capsid (CA) core nor interfere with reverse transcription (Fig 2). After completing reverse transcription, the viral pre-integration complex (PIC) enters the nucleus by passing through the nuclear pore and approaches the host genome. One intriguing finding in the current study was that POM121C (614–987) markedly reduces the amount of the integrated provirus form, but the 2-LTR form is reduced to a lesser extent (Fig 2A). This is reminiscent of the difference in 2-LTR production levels and integrated forms previously shown in Nup153-depleted cells, in which HIV-1 nuclear entry was blocked [18]. Perhaps POM121C (614–987) localized in the cytoplasm primarily impairs nuclear entry of HIV-1, and this may indirectly affect the proper intra-nuclear migration process and eventually limit integration. HIV-1 CA plays a pivotal role in nuclear import of viral DNA and this may potentially be a primary cause of POM121C (614–987)-mediated inhibition. Indeed, POM121C (614–987) inhibited HIV-1 but not MHIV-mMA12CA in dividing cells, suggesting that viral capsid defines the mode of nuclear entry and therefore determines whether POM121C (614–987) blocks infection or not. Accumulating evidence indicates that HIV-1 utilizes a particular pathway for nuclear entry that depends on CYPA, cleavage and polyadenylation-specific factor 6 (CPSF6) and NUP358, thereby evading innate immune sensors in the cytoplasm [42]. The HIV-1 capsid mutant N74D, which is not targeted by CPSF6, can use a different pathway for nuclear entry that does not involve CPSF6 nor NUP358, but is recognized by cytoplasmic innate immune sensors [44]. The present results that POM121C (614–987) inhibited infection with wild-type or N74D HIV-1 in a similar manner indicate that POM121C (614–987) suppresses both NUP358-dependent and -independent pathways (Fig 3B).

The production of similar amounts of the late RT product but marked decrease of the 2-LTR form of viral DNA in cells infected with the D116G mutant HIV-1 further support the idea that blockade by POM121C (614–987) occurs before integration. Taking these results together, we conclude that POM121C (614–987) inhibits HIV-1 replication after completion of reverse transcription and before the integration event.

To investigate how POM121C (614–987) blocks HIV-1 replication during nuclear import and intra-nuclear trafficking of HIV-1 DNA, we sought proteins binding POM121C (614–987) involved in HIV-1 inhibition. Binding of POM121C to viral components remains controversial. Fouchier *et al*. [45] reported yeast two-hybrid interaction of POM121 with Vpr, while Le Rouzic *et al*. [46] did not detect this interaction. Using immunoprecipitation and GST pull-down assays, we investigated whether POM121C interacts with Vpr but failed to detect any positive signal. Attention was therefore directed towards host cell factors that are targeted by POM121C (614–987). Because POM121C (801–987) proved to be sufficient to inhibit HIV-1 infection when fused with GST (Fig 4C), this fusion protein was used as bait. In this way, we have identified a number of candidates by MS at a good confidence level (Table 2). We selected KPNB1 for further study because this protein was found in the most prominent band 3 and had the highest number of matches. Importantly, the KPNB1 binding properties of POM121C mutants closely correlated with their level of HIV-1 blockade (Fig 6). The binding of POM121C mutants to KPNB1 is, however, not completely unexpected given that several previous studies demonstrated interactions between FG sequence repeats found in many nucleoporins and transport factors including KPNB1 [10, 47]. POM121C may belong to those nucleoporins that sequentially dock and undock with KPNB1/PIC complexes when HIV-1 PIC transits through nuclear pore complexes. Our attempts to determine whether endogenous full-length POM121C interacts with KPNB1 by means of immunoprecipitation experiments with two different antibodies specifically recognizing POM121C or KPNB1 were unsuccessful due in part to the limited expression of full-length POM121C in the nucleus. Thus, the possibility remains that endogenous POM121C physically interacts with KPNB1 under physiological conditions. To confirm the importance of endogenous POM121C or KPNB1 for HIV-1 replication, we had repeatedly attempted to knock-down POM121C or KPNB1 using shRNA, but unfortunately, depletion of these essential protein seriously affected the viability of cells, which made it impossible to assess HIV-1 infection. In future studies, it will be important to determine the exact role of POM121C and KPNB1 in the nuclear import and integration of HIV-1 DNA.

## Conclusions

Accumulating evidence indicates that the HIV-1 capsid is an essential determinant of nuclear translocation of viral cDNA and that many host cell factors including karyopherins and nuclear pore complex components mediate this process. We have shown in this article that an N-terminally truncated form of POM121C potently inhibits HIV-1 infection after completion of reverse transcription and before integration. This inhibition closely correlates with its binding to KPNB1, suggesting an important role for this karyopherin in HIV-1 replication.

## References

[1] Goff SP. Host factors exploited by retroviruses. Nat Rev Microbiol. 2007;5(4):253–63. Epub 2007/02/28. doi: 10.1038/nrmicro1541 . 17325726 10.1038/nrmicro1541

[2] Brass AL, Dykxhoorn DM, Benita Y, Yan N, Engelman A, Xavier RJ, et al Identification of host proteins required for HIV infection through a functional genomic screen. Science. 2008;319(5865):921–6. Epub 2008/01/12. doi: 10.1126/science.1152725 . 18187620 10.1126/science.1152725

[3] Hutten S, Walde S, Spillner C, Hauber J, Kehlenbach RH. The nuclear pore component Nup358 promotes transportin-dependent nuclear import. J Cell Sci. 2009;122(Pt 8):1100–10. Epub 2009/03/21. doi: 10.1242/jcs.040154 . 19299463 10.1242/jcs.040154

[4] Woodward CL, Prakobwanakit S, Mosessian S, Chow SA. Integrase interacts with nucleoporin NUP153 to mediate the nuclear import of human immunodeficiency virus type 1. J Virol. 2009;83(13):6522–33. Epub 2009/04/17. doi: 10.1128/JVI.02061-08 ; 19369352 10.1128/JVI.02061-08 PMC2698555

[5] Ciuffi A, Bushman FD. Retroviral DNA integration: HIV and the role of LEDGF/p75. Trends Genet. 2006;22(7):388–95. doi: 10.1016/j.tig.2006.05.006 16730094 10.1016/j.tig.2006.05.006

[6] Dharan A, Talley S, Tripathi A, Mamede JI, Majetschak M, Hope TJ, et al KIF5B and Nup358 Cooperatively Mediate the Nuclear Import of HIV-1 during Infection. Plos Pathog. 2016;12(6):e1005700 doi: 10.1371/journal.ppat.1005700 ; 27327622 10.1371/journal.ppat.1005700 PMC4915687

[7] Fassati A. HIV infection of non-dividing cells: a divisive problem. Retrovirology. 2006;3 Artn 74 doi: 10.1186/1742-4690-3-74 17067381 10.1186/1742-4690-3-74 PMC1635064

[8] Fahrenkrog B, Aebi U. The nuclear pore complex: Nucleocytoplasmic transport and beyond. Nat Rev Mol Cell Bio. 2003;4(10):757–66. doi: 10.1038/nrm1230 14570049 10.1038/nrm1230

[9] Paulillo SM, Phillips EM, Koser J, Sauder U, Ullman KS, Powers MA, et al Nucleoporin domain topology is linked to the transport status of the nuclear pore complex. J Mol Biol. 2005;351(4):784–98. doi: 10.1016/j.jmb.2005.06.034 16045929 10.1016/j.jmb.2005.06.034

[10] Tran EJ, Wente SR. Dynamic nuclear pore complexes: Life on the edge. Cell. 2006;125(6):1041–53. doi: 10.1016/j.cell.2006.05.027 16777596 10.1016/j.cell.2006.05.027

[11] Stewart M. Molecular mechanism of the nuclear protein import cycle. Nat Rev Mol Cell Bio. 2007;8(3):195–208. doi: 10.1038/nrm2114 17287812 10.1038/nrm2114

[12] Chin CR, Perreira JM, Savidis G, Portmann JM, Aker AM, Feeley EM, et al Direct Visualization of HIV-1 Replication Intermediates Shows that Capsid and CPSF6 Modulate HIV-1 Intra-nuclear Invasion and Integration. Cell Rep. 2015;13(8):1717–31. doi: 10.1016/j.celrep.2015.10.036 ; 26586435 10.1016/j.celrep.2015.10.036 PMC5026322

[13] Sowd GA, Serrao E, Wang H, Wang W, Fadel HJ, Poeschla EM, et al A critical role for alternative polyadenylation factor CPSF6 in targeting HIV-1 integration to transcriptionally active chromatin. Proc Natl Acad Sci U S A. 2016;113(8):E1054–63. doi: 10.1073/pnas.1524213113 ; 26858452 10.1073/pnas.1524213113 PMC4776470

[14] Yamashita M, Emerman M. Capsid is a dominant determinant of retrovirus infectivity in nondividing cells. J Virol. 2004;78(11):5670–8. Epub 2004/05/14. doi: 10.1128/JVI.78.11.5670-5678.2004 ; 15140964 10.1128/JVI.78.11.5670-5678.2004 PMC415837

[15] Lee K, Ambrose Z, Martin TD, Oztop I, Mulky A, Julias JG, et al Flexible use of nuclear import pathways by HIV-1. Cell Host Microbe. 2010;7(3):221–33. Epub 2010/03/17. doi: 10.1016/j.chom.2010.02.007 ; 20227665 10.1016/j.chom.2010.02.007 PMC2841689

[16] Ocwieja KE, Brady TL, Ronen K, Huegel A, Roth SL, Schaller T, et al HIV integration targeting: a pathway involving Transportin-3 and the nuclear pore protein RanBP2. Plos Pathog. 2011;7(3):e1001313 doi: 10.1371/journal.ppat.1001313 ; 21423673 10.1371/journal.ppat.1001313 PMC3053352

[17] Krishnan L, Matreyek KA, Oztop I, Lee K, Tipper CH, Li X, et al The requirement for cellular transportin 3 (TNPO3 or TRN-SR2) during infection maps to human immunodeficiency virus type 1 capsid and not integrase. J Virol. 2010;84(1):397–406. doi: 10.1128/JVI.01899-09 ; 19846519 10.1128/JVI.01899-09 PMC2798409

[18] Matreyek KA, Engelman A. The requirement for nucleoporin NUP153 during human immunodeficiency virus type 1 infection is determined by the viral capsid. J Virol. 2011;85(15):7818–27. doi: 10.1128/JVI.00325-11 ; 21593146 10.1128/JVI.00325-11 PMC3147902

[19] Truant R, Cullen BR. The arginine-rich domains present in human immunodeficiency virus type 1 Tat and Rev function as direct importin beta-dependent nuclear localization signals. Mol Cell Biol. 1999;19(2):1210–7. 9891055 10.1128/mcb.19.2.1210 PMC116050

[20] Kataoka N, Bachorik JL, Dreyfuss G. Transportin-SR, a nuclear import receptor for SR proteins. J Cell Biol. 1999;145(6):1145–52. ; 10366588 10.1083/jcb.145.6.1145 PMC2133142

[21] De Iaco A, Santoni F, Vannier A, Guipponi M, Antonarakis S, Luban J. TNPO3 protects HIV-1 replication from CPSF6-mediated capsid stabilization in the host cell cytoplasm. Retrovirology. 2013;10:20 Epub 2013/02/19. doi: 10.1186/1742-4690-10-20 ; 23414560 10.1186/1742-4690-10-20 PMC3599327

[22] Fassati A, Gorlich D, Harrison I, Zaytseva L, Mingot JM. Nuclear import of HIV-1 intracellular reverse transcription complexes is mediated by importin 7. EMBO J. 2003;22(14):3675–85. Epub 2003/07/11. doi: 10.1093/emboj/cdg357 ; 12853482 10.1093/emboj/cdg357 PMC165627

[23] Zielske SP, Stevenson M. Importin 7 may be dispensable for human immunodeficiency virus type 1 and simian immunodeficiency virus infection of primary macrophages. J Virol. 2005;79(17):11541–6. Epub 2005/08/17 doi: 10.1128/JVI.79.17.11541-11546.2005 ; 16103209 10.1128/JVI.79.17.11541-11546.2005 PMC1193637

[24] Mitchell JM, Mansfeld J, Capitanio J, Kutay U, Wozniak RW. Pom121 links two essential subcomplexes of the nuclear pore complex core to the membrane. J Cell Biol. 2010;191(3):505–21. Epub 2010/10/27. doi: 10.1083/jcb.201007098 ; 20974814 10.1083/jcb.201007098 PMC3003318

[25] Morita S, Kojima T, Kitamura T. Plat-E: an efficient and stable system for transient packaging of retroviruses. Gene Ther. 2000;7(12):1063–6. Epub 2000/06/29. doi: 10.1038/sj.gt.3301206 . 10871756 10.1038/sj.gt.3301206

[26] Planelles V, Bachelerie F, Jowett JBM, Haislip A, Xie YM, Banooni P, et al Fate of the Human-Immunodeficiency-Virus Type-1 Provirus in Infected-Cells—a Role for Vpr. Journal of Virology. 1995;69(9):5883–9. 7637036 10.1128/jvi.69.9.5883-5889.1995 PMC189467

[27] Hori T, Takeuchi H, Saito H, Sakuma R, Inagaki Y, Yamaoka S. A carboxy-terminally truncated human CPSF6 lacking residues encoded by exon 6 inhibits HIV-1 cDNA synthesis and promotes capsid disassembly. J Virol. 2013;87(13):7726–36. Epub 2013/05/10. doi: 10.1128/JVI.00124-13 ; 23658440 10.1128/JVI.00124-13 PMC3700264

[28] Masuda T, Planelles V, Krogstad P, Chen ISY. Genetic-Analysis of Human-Immunodeficiency-Virus Type-1 Integrase and the U3 Att Site—Unusual Phenotype of Mutants in the Zinc Finger-Like Domain. Journal of Virology. 1995;69(11):6687–96. 7474078 10.1128/jvi.69.11.6687-6696.1995 PMC189578

[29] Takeuchi H, Ishii H, Kuwano T, Inagaki N, Akari H, Matano T. Host cell species-specific effect of cyclosporine A on simian immunodeficiency virus replication. Retrovirology. 2012;9 Artn 3 doi: 10.1186/1742-4690-9-3 22225545 10.1186/1742-4690-9-3 PMC3311600

[30] Saitoh Y, Yamamoto N, Dewan MZ, Sugimoto H, Martinez Bruyn VJ, Iwasaki Y, et al Overexpressed NF-kappaB-inducing kinase contributes to the tumorigenesis of adult T-cell leukemia and Hodgkin Reed-Sternberg cells. Blood. 2008;111(10):5118–29. Epub 2008/02/29. doi: 10.1182/blood-2007-09-110635 . 18305221 10.1182/blood-2007-09-110635

[31] Sayah DM, Sokolskaja E, Berthoux L, Luban J. Cyclophilin A retrotransposition into TRIM5 explains owl monkey resistance to HIV-1. Nature. 2004;430(6999):569–73. Epub 2004/07/10. doi: 10.1038/nature02777 . 15243629 10.1038/nature02777

[32] Morita E, Sandrin V, Chung HY, Morham SG, Gygi SP, Rodesch CK, et al Human ESCRT and ALIX proteins interact with proteins of the midbody and function in cytokinesis. EMBO J. 2007;26(19):4215–27. Epub 2007/09/15. doi: 10.1038/sj.emboj.7601850 ; 17853893 10.1038/sj.emboj.7601850 PMC2230844

[33] Yamaoka S, Courtois G, Bessia C, Whiteside ST, Weil R, Agou F, et al Complementation cloning of NEMO, a component of the IkappaB kinase complex essential for NF-kappaB activation. Cell. 1998;93(7):1231–40. Epub 1998/07/10. . 9657155 10.1016/s0092-8674(00)81466-x

[34] Onishi M, Kinoshita S, Morikawa Y, Shibuya A, Phillips J, Lanier LL, et al Applications of retrovirus-mediated expression cloning. Exp Hematol. 1996;24(2):324–9. Epub 1996/02/01. . 8641361

[35] Yamamoto T, Tsunetsugu-Yokota Y, Mitsuki YY, Mizukoshi F, Tsuchiya T, Terahara K, et al Selective Transmission of R5 HIV-1 over X4 HIV-1 at the Dendritic Cell-T Cell Infectious Synapse Is Determined by the T Cell Activation State. Plos Pathog. 2009;5(1). ARTN e1000279 doi: 10.1371/journal.ppat.1000279 19180188 10.1371/journal.ppat.1000279 PMC2627922

[36] Higuchi M, Tsubata C, Kondo R, Yoshida S, Takahashi M, Oie M, et al Cooperation of NF-kappaB2/p100 activation and the PDZ domain binding motif signal in human T-cell leukemia virus type 1 (HTLV-1) Tax1 but not HTLV-2 Tax2 is crucial for interleukin-2-independent growth transformation of a T-cell line. J Virol. 2007;81(21):11900–7. Epub 2007/08/24. doi: 10.1128/JVI.00532-07 ; 17715223 10.1128/JVI.00532-07 PMC2168800

[37] Takeuchi H, Suzuki Y, Tatsumi M, Hoshino H, Daar ES, Koyanagi Y. Isolation and characterization of an infectious HIV type 1 molecular clone from a patient with primary infection. AIDS Res Hum Retroviruses. 2002;18(15):1127–33. Epub 2002/10/31. doi: 10.1089/088922202320567860 . 12402946 10.1089/088922202320567860

[38] Uno M, Saitoh Y, Mochida K, Tsuruyama E, Kiyono T, Imoto I, et al NF-kappaB inducing kinase, a central signaling component of the non-canonical pathway of NF-kappaB, contributes to ovarian cancer progression. PLoS One. 2014;9(2):e88347 Epub 2014/02/18. doi: 10.1371/journal.pone.0088347 ; 24533079 10.1371/journal.pone.0088347 PMC3922808

[39] Suzuki Y, Misawa N, Sato C, Ebina H, Masuda T, Yamamoto N, et al Quantitative analysis of human immunodeficiency virus type 1 DNA dynamics by real-time PCR: integration efficiency in stimulated and unstimulated peripheral blood mononuclear cells. Virus Genes. 2003;27(2):177–88. Epub 2003/09/23. . 14501196 10.1023/a:1025732728195

[40] Feng WY, Tanaka R, Inagaki Y, Saitoh Y, Chang MO, Amet T, et al Pycnogenol, a procyanidin-rich extract from French maritime pine, inhibits intracellular replication of HIV-1 as well as its binding to host cells. Jpn J Infect Dis. 2008;61(4):279–85. Epub 2008/07/26. . 18653969

[41] Brun S, Chaloin L, Gay B, Bernard E, Devaux C, Lionne C, et al Electrostatic repulsion between HIV-1 capsid proteins modulates hexamer plasticity and in vitro assembly. Proteins. 2010;78(9):2144–56. doi: 10.1002/prot.22729 20455269 10.1002/prot.22729

[42] Schaller T, Ocwieja KE, Rasaiyaah J, Price AJ, Brady TL, Roth SL, et al HIV-1 capsid-cyclophilin interactions determine nuclear import pathway, integration targeting and replication efficiency. Plos Pathog. 2011;7(12):e1002439 Epub 2011/12/17. doi: 10.1371/journal.ppat.1002439 ; 22174692 10.1371/journal.ppat.1002439 PMC3234246

[43] Liu C, Perilla JR, Ning J, Lu M, Hou G, Ramalho R, et al Cyclophilin A stabilizes the HIV-1 capsid through a novel non-canonical binding site. Nat Commun. 2016;7:10714 doi: 10.1038/ncomms10714 ; 26940118 10.1038/ncomms10714 PMC4785225

[44] Rasaiyaah J, Tan CP, Fletcher AJ, Price AJ, Blondeau C, Hilditch L, et al HIV-1 evades innate immune recognition through specific cofactor recruitment. Nature. 2013;503(7476):402–+. doi: 10.1038/nature12769 24196705 10.1038/nature12769 PMC3928559

[45] Fouchier RAM, Meyer BE, Simon JHM, Fischer U, Albright AV, Gonzalez-Scarano F, et al Interaction of the human immunodeficiency virus type 1 Vpr protein with the nuclear pore complex. Journal of Virology. 1998;72(7):6004–13. 9621063 10.1128/jvi.72.7.6004-6013.1998 PMC110405

[46] Le Rouzic E, Mousnier A, Rustum C, Stutz F, Hallberg E, Dargemont C, et al Docking of HIV-1 Vpr to the nuclear envelope is mediated by the interaction with the nucleoporin hCG1. J Biol Chem. 2002;277(47):45091–8. Epub 2002/09/14. doi: 10.1074/jbc.M207439200 . 12228227 10.1074/jbc.M207439200

[47] Bayliss R, Littlewood T, Strawn LA, Wente SR, Stewart M. GLFG and FxFG nucleoporins bind to overlapping sites on importin-beta. Journal of Biological Chemistry. 2002;277(52):50597–606. doi: 10.1074/jbc.M209037200 12372823 10.1074/jbc.M209037200

